# Sero-prevalence of hepatitis B virus and compliance with hepatitis B vaccination schedules among outpatient clinic attendees in Nairobi

**DOI:** 10.1371/journal.pone.0281256

**Published:** 2023-02-02

**Authors:** Benard Langat, Edward K. Muge, Doris Night, Fredrick Okoth, Kevin O. Ochwedo, Elijah M. Songok

**Affiliations:** 1 Department of Medical Biochemistry, University of Nairobi, Nairobi, Kenya; 2 Centre for Virus Research, Kenya Medical Research Institute, Nairobi, Kenya; 3 Faculty of Science and Technology, Department of Biology, University of Nairobi, Nairobi, Kenya; University of Cincinnati College of Medicine, UNITED STATES

## Abstract

**Background:**

Hepatitis B is becoming a growing public health problem in Kenya. To combat the threat, HBV vaccination should be recommended, particularly for individuals who are not covered by the national immunization program. Vaccination provides sero-protection rates approaching 95% among healthy adults after completing the three-dose vaccination course, but decreases to 87% among those who receive only two doses, emphasizing the importance of completing the three-dose vaccination course. However, data on adult adherence to HBV multi-dose vaccines in Sub-Saharan Africa are limited, despite the fact that this information is critical for prevention. As a result, more research on HBV vaccine dose completion is required. The purpose of this study is to estimate the prevalence of hepatitis B virus infection among out-patient clinic attendees in Nairobi, Kenya, as well as to identify beneficiaries of free vaccination and barriers to completing the recommended vaccine doses.

**Methods:**

Between July 30^th^ and September 30^th^, 2015, 2644 outpatient clinic attendees aged ≥ 4 were recruited from three hospitals in Nairobi County, Kenya: Mama Lucy, Riruta, and Loco. Self-administered questionnaires were used to collect socio-demographic information, and blood samples were tested for hepatitis B surface antigen (HBsAg) using the KEMRI HEPCELL Rapid® (Hepatitis B Detection kit) test kit. Individuals who tested negative for HBsAg were given a free course of three doses of HBV vaccine. The vaccination register provided information on the number of doses administered.

**Results:**

The average age of the study population was 31.4 years (range: 4–66), with females accounting for 59.2%. 1.82% (48/2644) of the participants tested positive for HBsAg. Among the 2596 individuals eligible for vaccination, 66% (1720/2596) received at least one dose, and 51.8% (1345/2596) received all three doses. Vaccination acceptance increased with age, with older patients more likely to return for subsequent dose (OR>1 for second and third dose). Unavailability and failure to contact client were cited as significant (p<0.0001) barrier to vaccination completion by 53.7% (666/1226, 95% CI 0.5–0.6) and 37% (454/1226, 95% CI 0.3–0.4) of respondents respectively.

**Conclusion:**

The prevalence of HBV infection among outpatient clinic attendees highlights the importance of expanding HBV immunization programs in Kenya. However, given the low vaccination completion rate, there is a need for public awareness of the vaccine’s importance in preventing HBV and HBV-related complications.

## Introduction

The global burden of hepatitis B virus (HBV) infection is estimated to be 290 million cases, the vast majority of which go undiagnosed and untreated [1, 2]. In terms of fatalities, approximately 887,000 deaths in 2015 were associated with two major HBV-related complications: cirrhosis and hepatocellular carcinoma (HCC) [3]. In Africa, robust HBV epidemiology data are lacking, and some populations are vulnerable due to poverty, stigma, and co-endemic human immunodeficiency virus (HIV) infection [2]. So far, a range of 6–20% HBV sero-prevalence has been reported in Africa [3], with East Africa recording an estimated HBsAg prevalence of 8% [4, 5]. In Kenya, the prevalence of HBsAg in the high-risk population is >8.0%% [5–7].

To combat HBV, the World Health Assembly approved a global health sector strategy to eliminate viral hepatitis as a public health threat by 2030, with a 90% reduction in new infections and a 65% reduction in mortality. According to the global strategy, eliminating hepatitis B disease requires collaboration across five core interventions, which include hepatitis B vaccination, prevention of HBV transmission from mother to child (PMTCT), blood and injection safety, harm reduction services for people who inject drugs, and increased testing and treatment [8]. To achieve these objectives, HBV immunization should be promoted and expanded to include other populations. The Kenya Expanded Programme on Immunization (KEPI) includes HBV vaccination as part of a universal vaccination strategy for all infants; the vaccine is also administered selectively to individuals considered to be at high risk [9]. Kenya had an estimated HB vaccination coverage rate of 78% in 2015, which increased to 87% in 2021 [10]. Vaccination against hepatitis B infection has been shown to be both clinically necessary and cost effective [11, 12], with sero-protection rates of about 95% among healthy adults after receiving the entire course of HBV vaccination [12–14], but decreasing up to 87% among those who received only two doses [15], emphasizing the importance of completing the three-dose vaccination course [3, 16]. The majority of studies on completion of multi-dose vaccine schedules have been conducted in pediatric and adolescent populations [17–19]. There have been few studies of series completion for multi-dose vaccinations in broad general adult populations [20], justifying the need for more research in this area.

In this context, pre-vaccination surveys were conducted to determine the prevalence of HBV among patients attending selected Nairobi hospitals. The study also documented this population’s HBV vaccination compliance rates and uncovered the factors associated with vaccine series incompletion.

## Materials and methods

### Study design and sampling

The study used three major recruitment strategies for participants: hospital sensitization via posters, oral announcements by health care workers, and contact with community leaders who organized recruitment days. This was a cross-sectional study in which 2644 patients were purposively recruited from the outpatient departments of three health care centers in Nairobi County, Kenya (Mama Lucy, Riruta, and Loco hospitals). The enrolled patients were attending routine clinics at the selected hospitals and were followed up on for a 6-month period after recruitment (August 1^st^, 2014 and May 2015). These hospitals are public health facilities that provide health care services at a reduced cost, allowing everyone, regardless of socioeconomic status, to access medical services. Individual vaccination compliance improves when large-scale, multi-site hepatitis B vaccination programs are used [21], hence our choice of three sites. Following the provision of oral consent, all participants were asked to complete a self-administered questionnaire in order to obtain socio-demographic information.

**Fig 1:**
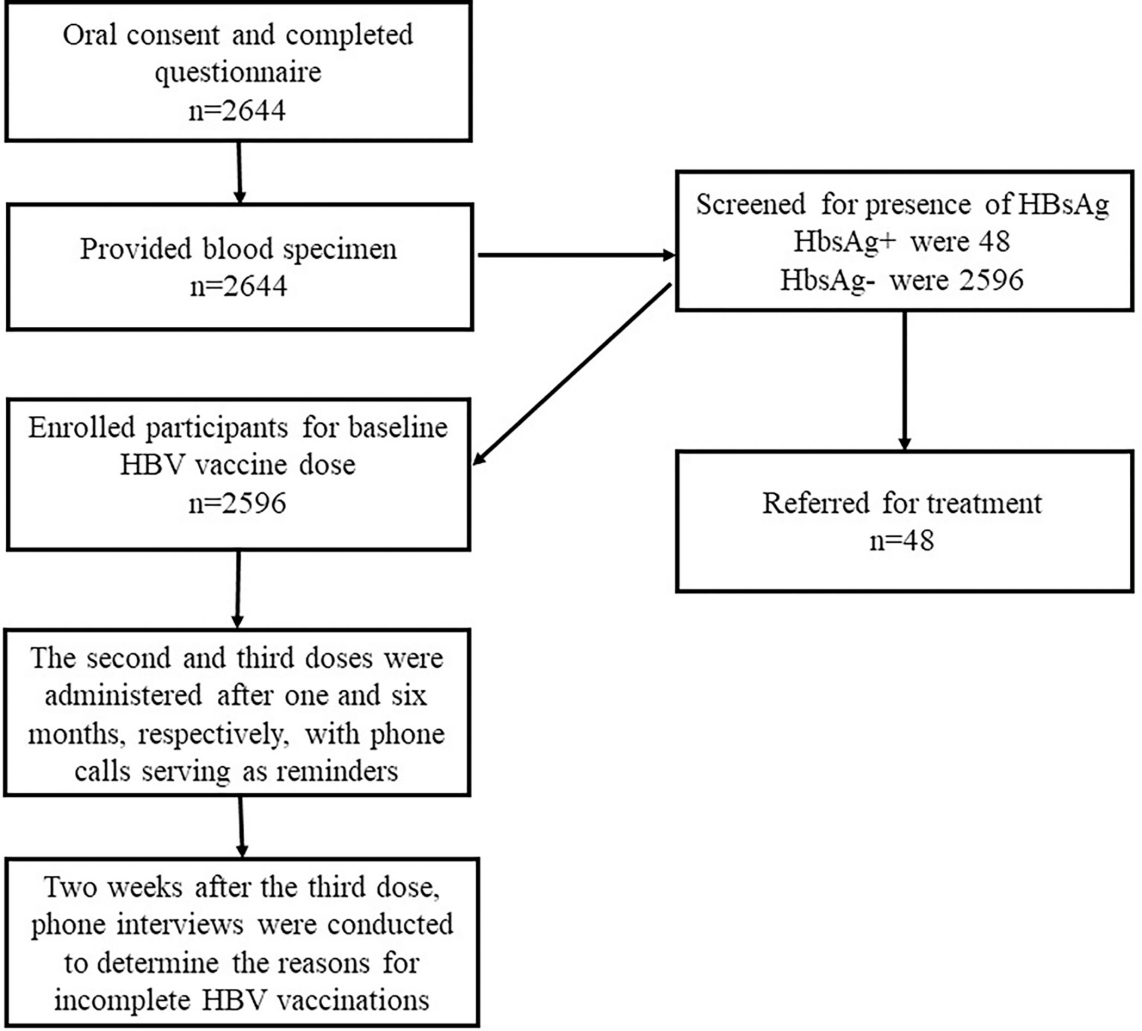
Sampling diagram of enrolled participants for hepatitis B three-dose series vaccine compliance from the outpatient departments of three Nairobi County health care centers.

All participants were tested for the presence of HBsAg by qualified medical laboratory technicians using the KEMRI Hep-cell kit according to the manufacturer’s instructions. The test kit has sensitivity of >98% and specificity of >95% [22, 23]. Participants were divided into two groups based on their screening results: those who were infected (HbsAg+) and those who were not (HbsAg-). Patients who tested negative for HbsAg were deemed eligible for vaccination and were given a free course of three doses of hepatitis B vaccine (**Fig 1**). Individuals who tested positive for HBsAg were notified and referred for treatment. HBV vaccination was administered in three doses via intramuscular injection. The first dose was the baseline dose, and the second and third doses were given one and six months later, respectively.

The vaccination register provided information on the number of doses received by each participant. This study used reminder phone calls by health care workers to improve the rate of return for the second and final dose, as demonstrated by Sellors et al., [24]. Two weeks after the last vaccination, the investigators conducted a phone interview with participants on a dedicated phone line to obtain reasons for incomplete HBV vaccination.

### Ethics approval

Ethical approval for the research was obtained from Kenya Medical Research Institute’s ethical review committee, (SERU 2209). All participants provided oral consent.

### Statistical analysis

Consistently and completely filled-out questionnaires, laboratory results, and phone interview data were entered in Microsoft Excel version 2016 before being imported into and analyzed with the Statistical Package for the Social Sciences (SPSS for Windows, version 17.0, SPSS Inc, and Chicago, IL, USA). To summarize the population under study, descriptive statistics such as sum, mean, standard deviation, standard error, and 95% confidence interval were computed. Multiple mean comparisons among and between variables were carried out using Chi-square and One-Way ANOVA test, followed by the Turkey HSD comparison test. Binary logistic regression models were used to determine the association between second and third vaccine dose acceptance and age grouping. Statistical significance was considered when p<0.05.

## Results

The 2644 participants who agreed to take part in the study ranged in age from 4 to 66 years old (average age of 31.4). Females made up 59.1% (1562/2644) of the total, while males made up 40.9% (1082/2644). A total of 48 (1.8%) people tested positive for HBsAg, with no significant difference in sero-prevalence across gender (χ^2^  = 2.519, df1, p = 0.112), and age group as determined by one-way ANOVA (*F* (6,2637) = 1.100, p = 0.359)) (**Table 1**). Male participants (2.3%, 25/1082) were more likely than females (1.5%, 23/1562) to be HBV seropositive (OR = 1.58, 95% CI 0.90–2.82).

**Table 1:** HBV prevalence according to age among outpatient clinic attendees in Kenya in 2015.

Parameter		Sample size	Seropositivity n (%, 95% CI)	P-value
Name	Level			
**Gender**	Female	1562	23 (1.5, 0.009–0.021)	0.112
	Male	1082	25 (2.3, 0.014–0.032)	
**Age group**	4–17	36	0 (0)	0.359
	18–25	586	10 (1.7, 0.006–0.027)	
	26–35	908	21 (2.3, 0.013–0.033)	
	36–45	556	10 (1.8, 0.007–0.029)	
	46–55	394	3 (0.8, -0.001–0.016)	
	56–65	123	4 (3.3, 0.001–0.064)	
	66–90	41	0 (0)	

Of the 2644 participants, 98.2%, or 2596 (all HbsAg-negative patients), expressed interest in receiving the first dose of vaccine and were subsequently vaccinated. Only 1720 (66.3%) of the 2596 vaccinated patients returned for a second shot, whereas 1345 (51.8%) received all three doses of the vaccine (**Table 2**). The number of people seeking second and third vaccination doses decreased significantly (χ^2^  = 112, df1, p<0.0001). Females (31.3%, 813/2596) completed the three-dose series more than males (20.5%, 532/2596) (OR = 1.1, 0.94–1.29). The difference in vaccination completion rates between male and female was not significant (2 = 1.563, df1, p = 0.211). Hepatitis B compliance was highest in patients aged ≥36 years, despite overall low compliance (**Table 2**). There was a significant difference *F* ((6, 2589) = 11.30, p<0.0001)) in compliance to second dosage between age groups, with age group 66–90 having higher compliance of 80.5% (33/41, 95% CI 0.7–0.9). Post hoc comparisons using Turkey HSD test revealed a significant difference (p<0.001) in seeking second dosage to be between age groups 18–25 verses 26–35, 18–25 vs. 46–55, 18–25 vs. 56–65, and 18–25 vs. 66–90. This was also observed among patients seeking third vaccination, with a significant difference *F* ((6, 2589) = 11.95, p<0.0001)) between age groups 18–25 vs. 36–45, 18–25 vs. 46–55, 18–25 vs. 56–65, 26–35 vs. 36–45, 26–35 vs. 46–55, and 26–35 vs. 56–65 revealed by post hoc comparisons using Turkey HSD test. In contrast to compliance with the second dosage, the majority of individuals who complied with the third dosage were between the ages of 56 and 65 (64.7%, 77/119, 95% CI 0.6–0.7). Most were more likely (OR = 1.8, p<0.0001) to stick to the second immunization than the third. Adherence to second and third vaccinations increased with age, with older age groups more likely to accept second and follow-up vaccinations (**Table 2**).

**Table 2:** HBV vaccination compliance rates according to ages among outpatient clinic attendees in Kenya in 2015.

Parameter		1^st^ dose n (%)	2^nd^ dose n (%)	OR (95% CI, P-value)	3^rd^ dose n (%)	OR (95% CI, P-value)
Age group	Vaccine candidates					
4–17	36	36 (100%)	25 (69.4%)	1	16 (44.4%)	1
18–25	576	576 (100%)	309 (53.6%)	0.5 (0.2–1.1, 0.069)	245 (42.5%)	0.9 (0.5–1.8, 0.822)
26–35	887	887 (100%)	584 (65.8%)	0.8 (0.4–1.7, 0.655)	413 (46.6%)	1.1 (0.6–2.1, 0.803)
36–45	546	546 (100%)	390 (71.4%)	1.1 (0.5–2.3, 0.799)	327 (59.9%)	1.9 (0.9–3.7, 0.072)
46–55	391	391 (100%)	290 (74.2%)	1.3 (0.6–2.7, 0.538)	241 (61.6%)	2 (1–4, 0.047) ^⁕^
56–65	119	119 (100%)	89 (74.8%)	1.3 (0.6–3, 0.525)	77 (64.7%)	2.3 (1.1–4.9, 0.032) ^⁕^
66–90	41	41 (100%)	33 (80.5%)	1.8 (0.6–5.2, 0.265)	26 (63.4%)	2.2 (0.9–5.4, 0.097)
**TOTAL**	**2596**	**2596 (100%)**	**1720 (66.3%)**	**-**	**1345 (51.8%)**	**-**

Among the 1226 patients who did not complete the vaccination series, the reasons given for non- compliance include unavailability at 53.7% (658/1226, 95% CI 0.5–0.6), 37% (454/1226, 95% CI 0.3–0.4) of client were not contacted, forgetting despite being sent message was 2.2% (27/1226, 95% CI 0.01–0.03), late for previous dose, thus affecting subsequent vaccinations was 1.6% (20/1226, 95% CI 0.01–0.02), went back to school were 1.3% (16/1226, 0.01–0.02), client not picking calls were 1.2% (15/1226, 95% CI 0.01–0.02), due to pregnancy were 0.9% (11/1226, 95% CI 0.003–0.01) and varied other reasons were collectively 2% (25/1226) (Table 3). The was a significant difference between the given reasons *F* ((18, 23275) = 842.5, p<0.0001)), with post hoc comparisons using Turkey HSD test revealing a significant difference on unavailability against all other reasons as well as client not contacted against the rest (p<0.0001).

**Table 3:** Reasons for incomplete HBV vaccination among outpatient clinic attendees in Kenya in 2015.

Reasons	Frequency n (%)	P-value
Unavailable	658 (53.7%) ^a^	<0.0001
Client not contacted	454 (37.0%) ^b^	
Forgot, despite being sent message	27 (2.2%) ^c^	
Late for previous dose, thus affecting subsequent vaccinations	20 (1.6%) ^c^	
Went back to School	16 (1.3%) ^c^	
Client not picking calls	15 (1.2%) ^c^	
Pregnant	11 (0.9%) ^c^	
Was sick	5 (0.4%) ^c^	
Was on booster	4 (0.3%) ^c^	
Lost direction to place of vaccination	4 (0.3%) ^c^	
Lack of fare to the Centre	3 (0.2%) ^c^	
Told medication was out of stock	2 (0.2%) ^c^	
Queue was long, thus gave up	1(0.1%) ^c^	
Parent not aware of exercise	1 (0.1%) ^c^	
Permission not provided from work place	1 (0.1%) ^c^	
Claims young to continue with exercise	1 (0.1%) ^c^	
Deceased	1 (0.1%) ^c^	
Pain	1 (0.1%) ^c^	
Venue of exercised locked	1 (0.1%) ^c^	
**TOTAL**	**1226 (100%)**	**-**

Different letter superscripts (^a, b,^ and ^c^) indicate that the values in each of the reasons raised by participants differ significantly.

## Discussion

The HBV prevalence observed in this study population (1.82%) is comparable to the estimated HBV prevalence in Kenyan populations (2–13%) from previous studies [5–7, 25–28]. In Eastern African countries, prevalence estimates range from 6–8%. Tanzania and Ethiopia, for example, have general population prevalence rates of 6 and 8%, respectively [29, 30], which are higher than the results of this study. As observed is previous study in Kenya, more HBV infections were recorded in males as compared to females [6, 31]. Furthermore, the current study set out to determine the acceptance and compliance of hepatitis B vaccination among the study population. The acceptance rate of 98.2% for the first dose among outpatient clinic attendees from various backgrounds was especially encouraging. One possible explanation for the observed high acceptance rate could be the offer of free vaccination. Lower rates of acceptance have previously been reported in studies from other countries, ranging from 23% to 69% [32, 33], indicating that this study was successful. The second reason for vaccination’s near-complete acceptance rate can be attributed to the fruitful efforts of health care workers who mobilized and encouraged patients to participate in the exercise. However, predictors of compliance may differ significantly from predictors of acceptance [34]. This is evidenced by a drop in the number of participants to 66.3% who received at least one dose of the vaccine, a figure lower than the 74% reported in a population of hard-to-reach individuals from homeless and outreach centers across London [35].

This study found that 51.7% of people received the entire HBV vaccination series. This is comparable to a recent study [35] that found that completion of all three vaccination shots can still be lower than expected, even when free vaccinations are provided. When compared to a recent study conducted among Kenyan health care students, the acceptance and completion rates were higher (51.7% versus 20.2% and 98.2% versus 85.8%, respectively) [36]. In contrast to the findings of this study, other studies among a low-income minority population [37], adults in the United Kingdom [38], and men in Republic of Korea [39] found lower three-dose series completion rates of 31%, 22% and 33% respectively, despite the vaccinations being provided free of charge in some countries.

The study found that the rate at which participants completed the vaccination series increased significantly with age, with patients aged ≥36 years more likely to complete the series than younger patients. This is consistent with previous research, which found that older age was a predictor of vaccination acceptance and completion among vulnerable populations [40, 41]. This is supported further by a recent study that found the highest compliance in patients aged 41 to 60 years [42]. These findings, however, contradict previous research that found age to be inversely related to adult vaccination status [43–46].

With more participants (53.1%) citing time as a constraint due to a hectic schedule, this study identified unavailability of participants or lack of time as the primary barrier to completing the entire vaccination series in Kenya. This is the first study to report unavailability as a barrier to completion of three dose series of HBV vaccination in general population in Kenya. The results are additional to previous findings by Maina and Bii [36] in which unavailability of vaccines was listed as a major barrier among Kenyan health care students. The findings however corroborates previous research by Juon et al. [47] conducted among Asian American in Baltimore-Washington Metropolitan area. Other minor reasons identified by this study included: participants who were not contacted (36.7%), and participants who forgot despite being sent a message (2.3%). The latter two barriers point to a lack of adequate follow-up by health care workers, confirming that HCW involvement may significantly impact on vaccination completion [16, 48, 49]. Barriers of less than 1% add to previous findings that the media, friends, and relatives do not serve as credible sources of vaccination information [16, 43, 48, 50].

The low vaccination compliance observed in this study necessitates a shift in strategy among public health stakeholders. Several strategies have been proposed to improve compliance among difficult-to-reach at-risk groups, including incentive schemes, outreach activities, reminder systems, and alternative vaccine schedules [51]. A shortened schedule of 0, 10, and 21 days, for example, has been shown to result in protective antibody levels in 90% of vaccinated individuals one year after primary vaccination [52, 53]. Importantly, there is evidence that accelerated schedules can improve HBV vaccination uptake, particularly among high-risk groups with whom it is difficult to maintain contact [51, 54, 55], while also being safe and effective [56–58]. Due to budget constraints, not all markers of HBV infection could be measured. As a result, the study was unable to distinguish between acute and chronic HBV infections, as well as between individuals who were immune to HBV and those who were still susceptible to HBV infection. The study also did not assess motivation toward completion of the three-dose series by compliant participants.

## Conclusion

The presence of HBV serological markers in unvaccinated study participants emphasizes the need for population-level preventative measures. However, adherence to and completion of the recommended multi-dose schedules for HBV are low in out-patient clinic attendees. In the latter case, the observed low rates of compliance provide an opportunity to raise awareness of existing compliance gaps.

## Supporting information

S1 File (XLSX)
